# Supplementary material for: Anti‐tumor efficacy of a novel CLK inhibitor via targeting RNA splicing and MYC‐dependent vulnerability
Source: EMBO Mol Med. 2018 May 16;10(6):e8289. doi: 10.15252/emmm.201708289 (PMC5991599; doi:10.15252/emmm.201708289)
Supplement: Supplementary file 1 — Appendix [file EMMM-10-e8289-s001.pdf]

**Appendix**

**Inventory of Appendix**

**Appendix Figure S1**

**Appendix Figure S2**

**Appendix Figure S3**

**Appendix Figure S4**

**Appendix Figure S5**

**Appendix Figure S6**

**Appendix Figure S7**

**Appendix Figure S8**

**Appendix Figure S9**

**Appendix Supplemental Methods**

**Appendix References**

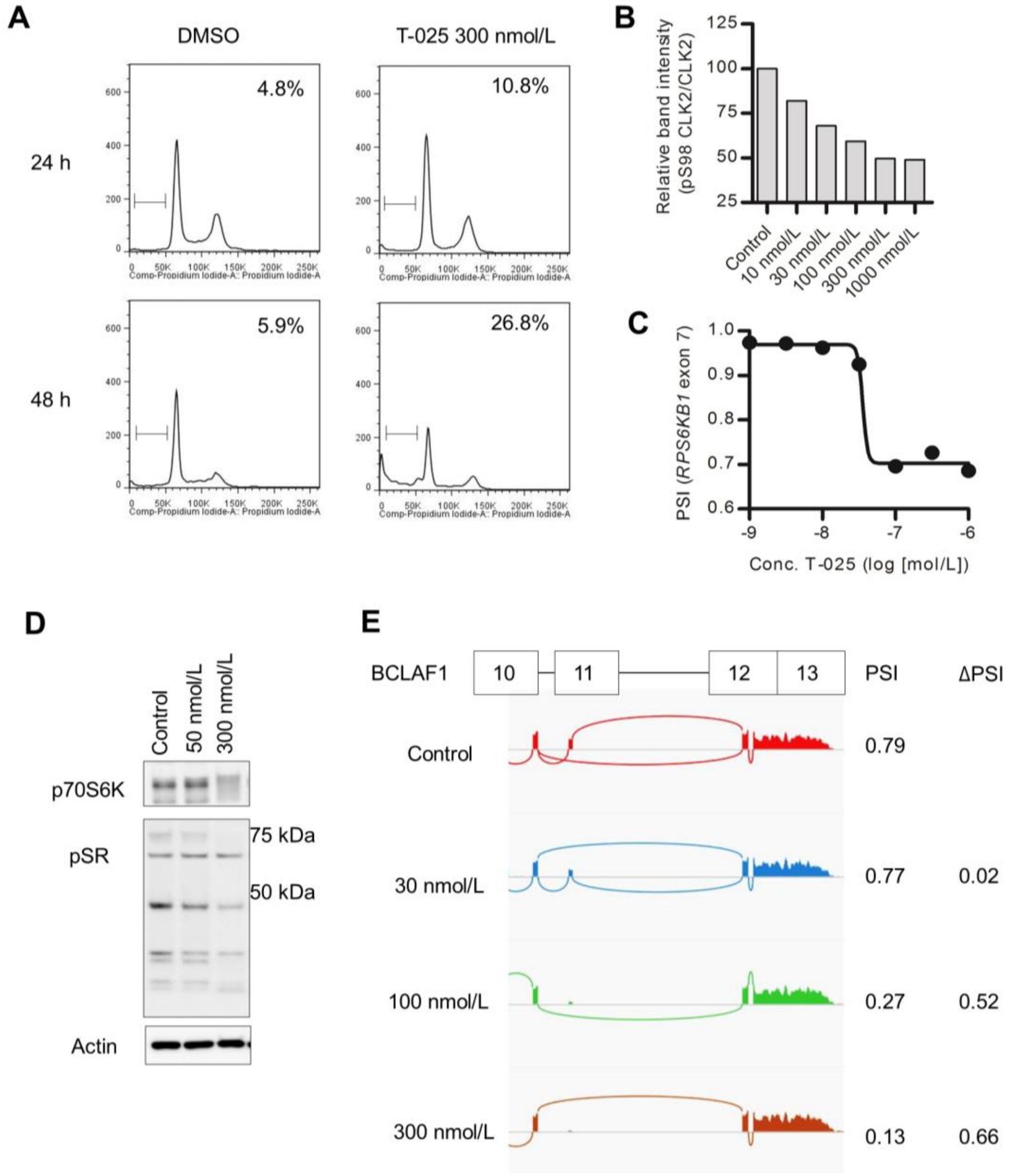


**Appendix Figure S1.** Additional data of *in vitro* mechanism of action of T-025 in MDA-MB-468 cells.

A, Cell cycle profile of MDA-MB-468 cells treated with T-025 at 300 nmol/L was analyzed using FACS. Percent of Sub-G1 population in each sample is shown ($n$ = 1).

B, Band intensities of pS98 CLK2 and CLK2 shown in Fig 2A were quantified and the

pS98 CLK2/CLK2 ratio was calculated.

C, Exon 7 skipping of *RPS6KB1* was induced by T-25. MDA-MB-468 cells were treated with T-025 for 6 hours. *RPS6KB1* exon 6–7 and 6–8 transcripts were measured by quantitative RT-PCR and the PSI value of each sample was calculated.

D, Cell lysate from MDA-MB-468 cells treated with T-025 for 48 hours was analyzed by immunoblotting with antibodies .

E, Sashimi plot of *BCLAF1* exon 11 and its PSI value in MDA-MB-468 treated with T-025 are shown.

**A**

| | | Non-treatment | | | T-025 300 nmol/L | | |
|---|---|---|---|---|---|---|---|
| | | Intensity | | Ratio | Intensity | | Ratio |
| Sequence | Position | Non-phos | Phos | P/Non-P | Non-phos | Phos | P/Non-P |
| RED**S**YHVR | S50 | 2.27E+06 | 9.13E+06 | 4.02 | 1.39E+06 | 2.90E+06 | 2.09 |
| GDAYYDTDYRH**S**YEYQT | S98 | 4.51E+03 | 1.56E+06 | 346 | 3.12E+03 | 2.31E+05 | 74 |
| AK**S**VEDDAEGHLIYHVGDWLQER | S142 | ND | 8.28E+05 | | ND | ND | |
| VVDFGSA**T**FDHEHHSTIVSTR | T332 | 2.74E+07 | 1.39E+07 | 0.507 | 9.97E+06 | 1.40E+06 | 0.140 |

**B**

YYDTDYRH**S**YEYQRENSSYRSQRSSRRKHRR

90 98 120

CLK2 peptide: DYRH_**S**YEYQRENSSY
pCLK2 peptide: DYRH**pS**YEYQRENSSY

**C**

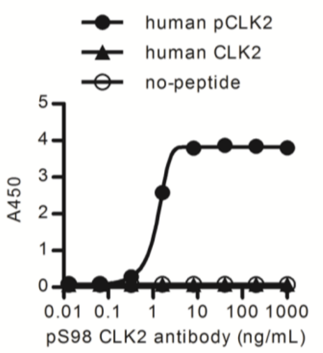


**D**

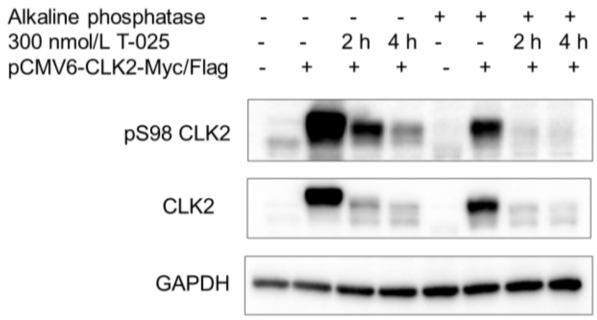


**Appendix Figure S2**. Phospho-specific antibody against CLK2 at Ser98.

A, The peptide detected by MS of IP samples from cell lysates of 293T cells expressing FLAG-CLK2. Bold characters indicate phosphorylated sites. 293T cells transfected with FLAG-CLK2 were treated with or without 300 nmol/L T-025 for 2 hours. FLAG-CLK2 was immunoprecipitated from total cell lysate using an anti-FLAG-antibody.

B, Amino acid sequence of CLK2 residues 90–120. The 15 underlined amino acids were used as an antigen.

C, Binding activity of the anti-pS98 CLK2 antibody against phosphorylated and

non-phosphorylated CLK peptide was measured. The anit-pS98 CLK2 antibody bound only to the CLK2 peptide with phospho-Serine98.

D, Cell lysate from 293T cells transfected with FLAG-tag CLK2 and treated with or without T-025 were incubated with alkaline phosphatase for 1 hour and analyzed with phospho-specific antibodies.

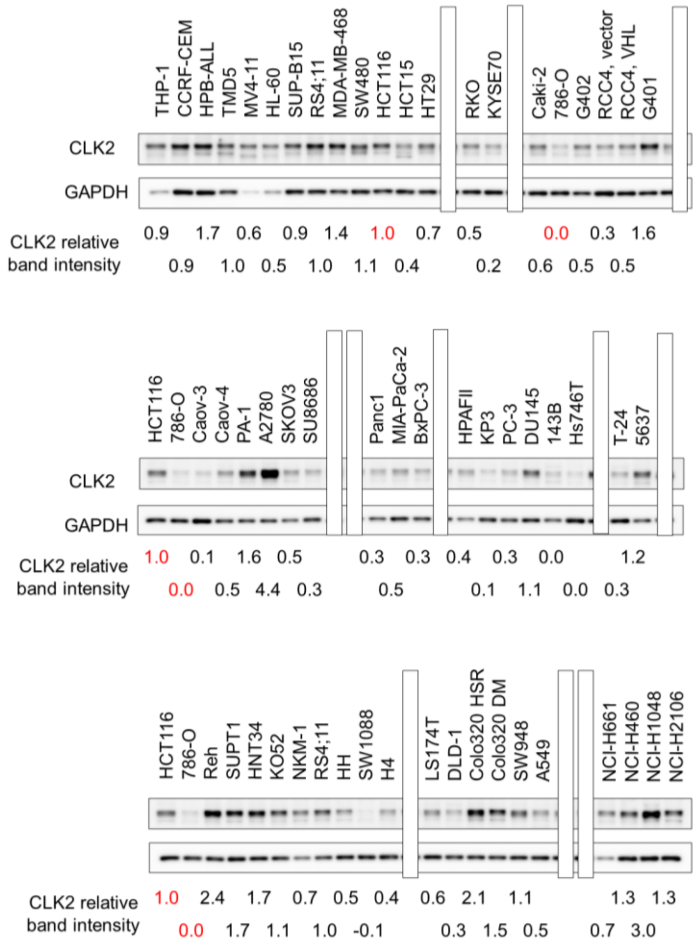


**Appendix Figure S3.** Expression level of CLK2 in various cancer cell lines.

The expression level or CLK2 in various cancer cell lines were analyzed by immunoblotting. Also shown relative band intensity to HCT116 and 786-O (Red). 11 out of 66 cell lines were removed from this manuscript due to no validation of negative status in mycoplasma test.

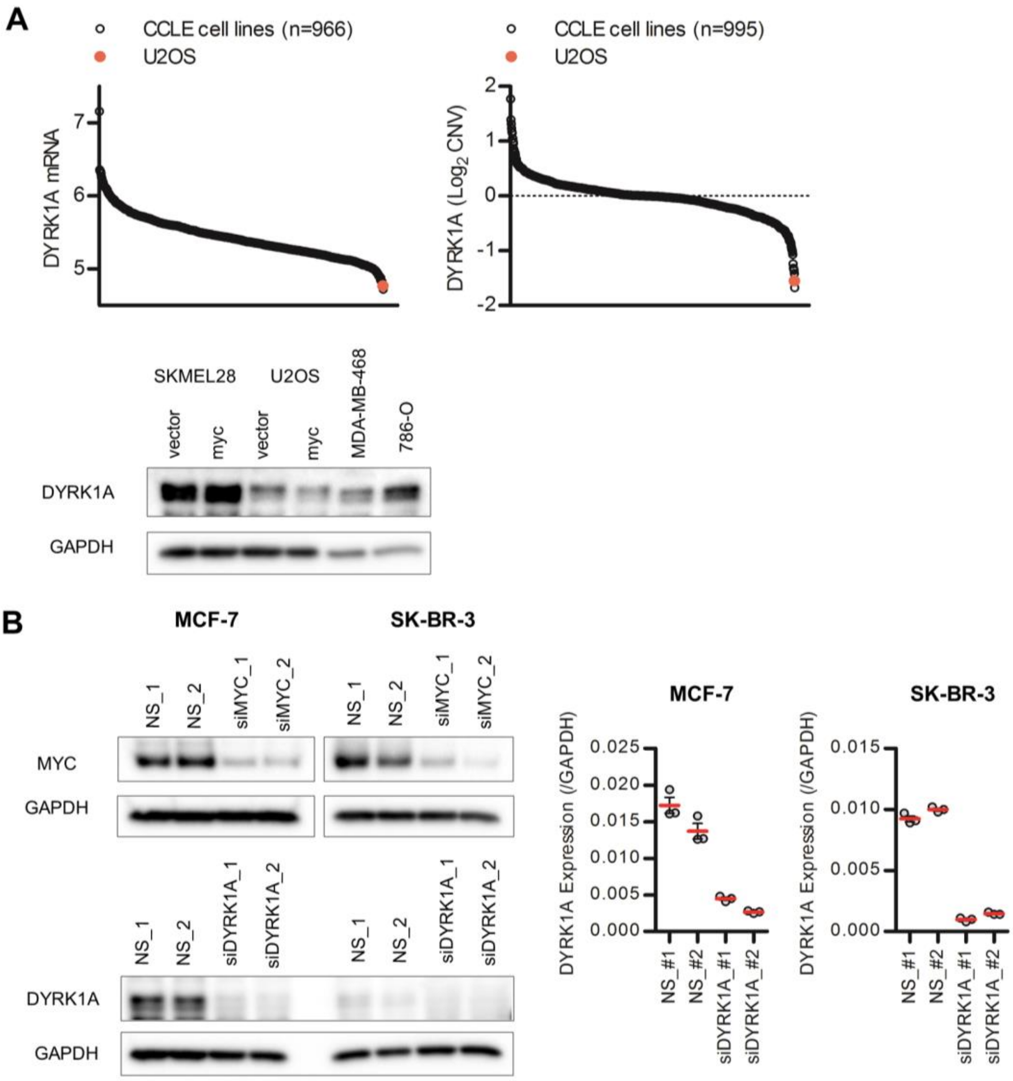


**Appendix Figure S4.** Expression level of MYC and DYRK1A in cell lines.

A, The expression level or CNV of DYRK1A in cancer cell lines of CCLE database. Red circle shows U2OS cells. DYRK1A protein level of MYC-inducible SK-MEL-28 or U2OS cell were measured by immunoblotting.

B, MYC or DYRK1A protein levels at 72 h or DYRK1A mRNA levels at 48 h after transfection in SKBR3 or MCF7 breast cancer cells were measured by immunoblotting

or RT-PCR.

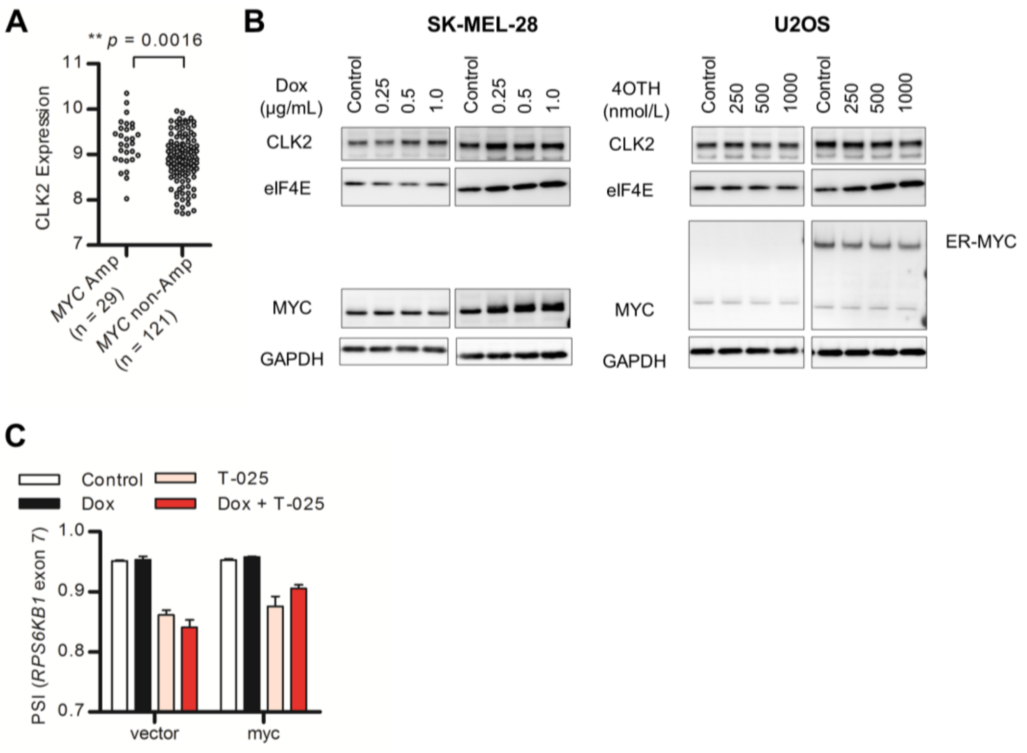


**Appendix Figure S5.** Additional data of MYC-inducible cell lines

A, CLK2 expression level of the *MYC*-amplified ($n$ = 29) and other solid cancer cell lines ($n$ = 121) described in Fig 3B are shown.

B, Protein level of CLK2 in SK-MEL-28 or U2OS cells treated with Dox or 4OTH for 72 h were analyzed by immunoblotting. Protein level of eIF4E, a known downstream target of MYC, was shown as a positive control.

C, Exon 7 skipping of *PRS6KB1* was induced by T-25. MYC-inducible SK-MEL-28 cells pretreated with Dox were treated with 100 nmol/L T-025 for 6 h. *PRS6KB1* exon 6–8 and 6–8 transcripts were measured by quantitative RT-PCR and the PSI value of each sample was calculated.

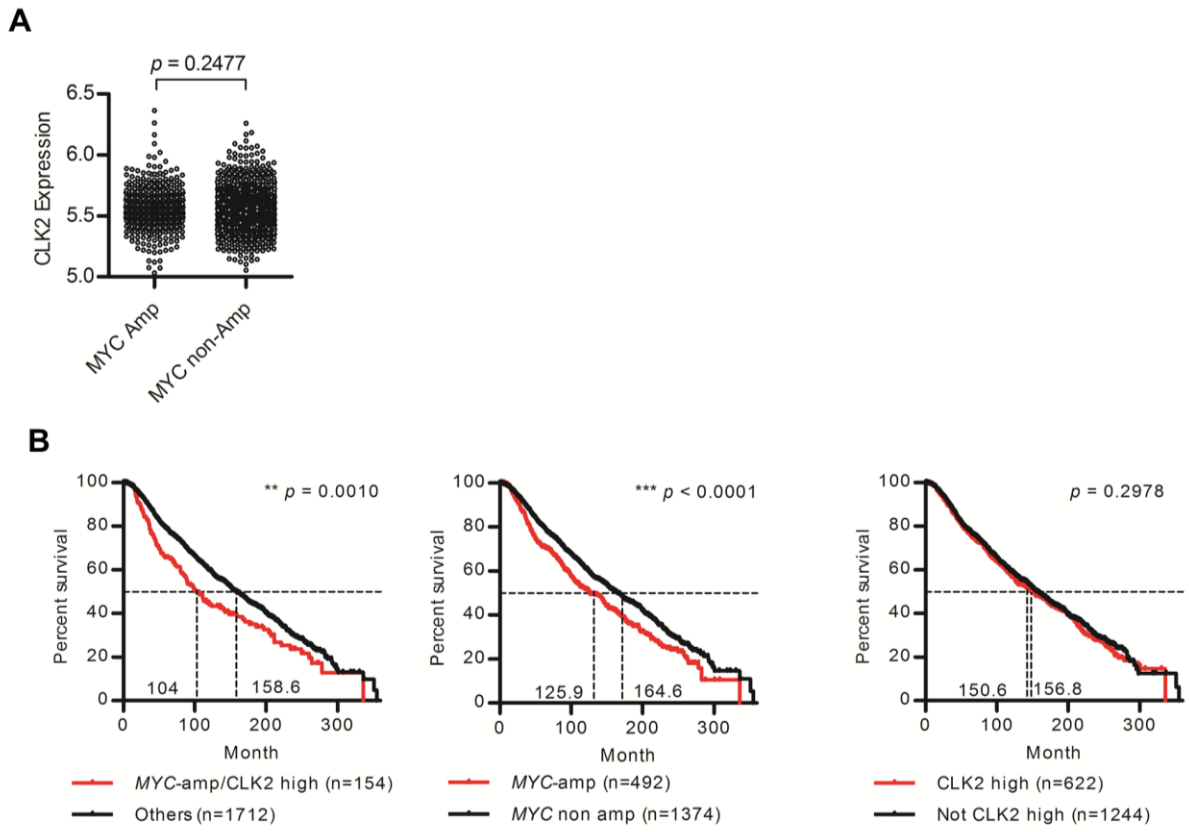


**Appendix Figure S6.** Additional data from patient database

A, Correlation of CLK2 expression level with its *MYC*-amplified status in the clinical samples. A Mann-Whitney test was performed.

B, Kaplan-Meier survival curve of breast cancer patients. Patients were divided into two groups by *MYC*-amplified status and expression level of CLK2. Median survival time of each group was calculated by Prism and a Log-rank test was performed.

A

| X-Ray Structure | CLK2-T-025/PDB CODE: 5UNP |
|---|---|
| **Data collection** | |
| Resolution range (Å) | 50–2.92 (2.97–2.92) |
| Space group | $I4_1$ |
| Cell dimensions: | |
| *a*, *b*, *c* (Å) | 141.252, 141.252, 124.370 |
| a, b, g (° ) | 90.000, 90.000, 90.000 |
| $R_{sym}$ | 5.9 (100.0) |
| <I / sI> | 24.0 (1.4) |
| Completeness (%) | 99.8 (100.00) |
| Redundancy | 5.0 (5.0) |
| **Structure Refinement** | |
| Resolution (Å) | 38.97-2.92 |
| No. reflections | 25,181 |
| No. reflections Rfree test set | 1,333 |
| Rwork / Rfree | 20.1/ 23.4 |
| No. atoms: | |
| Protein | 5,762 |
| Ligand | 58 |
| Waters | 5 |
| Mean B Value ($Å^2$) | 106.0 |
| Protein B Value ($Å^2$) | 107.0 |
| Ligand B Value ($Å^2$) | 93.5 |
| Water B Value ($Å^2$) | 70.2 |
| RMS Bond lengths (Å) | 0.007 |
| RMS Bond angles (° ) | 1.177 |
| Ramachandron statistics: | |
| Favored (%) | 91.45 |
| Outliers (%) | 0.29 |

*Values in parentheses are for highest-resolution shell.

B

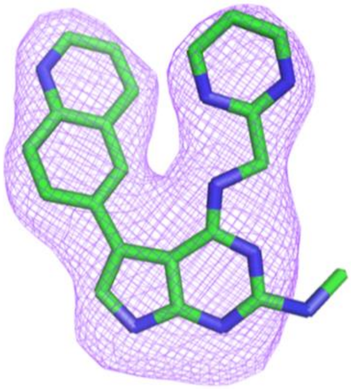

**Appendix Figure S7.** Additional X-ray crystallography data of CLK2 and T-025.

A, X-ray data collection and refinement statistics.

B, Omit electron density Fo-Fc map (rendered as a mesh) was generated by omitting atoms corresponding to the compound (rendered as green sticks). The map is contoured at ± 3.0 σ. The figure was generated using Pymol.

| Fig 5C | $IC_{50}$ (95% CI) |
|---|---|
| SK-MEL-28 vector | 136 (130-142) |
| SK-MEL-28 vector + Dox | 125 (121-129) |
| SK-MEL-28 myc | 125 (121-130) |
| SK-MEL-28 myc + Dox | 65.0 (62.3-67.7) |

| Fig 5G | $IC_{50}$ (95% CI) |
|---|---|
| U2OS vector | 129 (118-140) |
| U2OS vector + Dox | 104 (99.2-119) |
| U2OS myc | 103 (94.4-112) |
| U2OS myc + Dox | 46.8 (41.7-52.3) |

| Fig 5I | $IC_{50}$ (95% CI) | |
|---|---|---|
| | MCF7 | SKBR3 |
| NS_1 | 326 (293-366) | 84.3 (80.1-89.0) |
| NS_2 | 284 (251-324) | 82.8 (77.9-88.3) |
| siMYC_1 | 627 (555-702) | 158 (143-176) |
| siMYC_2 | 489 (447-536) | 156 (133-188) |
| siDYRK1A_1 | 269 (229-324) | 64.6 (61.9-67.5) |
| siDYRK1A_2 | 287 (261-617) | 90.9 (85.8-96.6) |

**Appendix Figure S8.** $IC_{50}$ values of T-025 in MYC-inducible cell lines.

$IC_{50}$ values and 95% confidence intervals (95% CIs) of T-025 in the MYC-inducible cell lines (Fig 5C and 5D) or breast cancer cell lines pre-treated with siRNAs (Fig 5I) were calculated by using Prism 5.0.

Statistical analysis below were performed by using GraphPad Prism 5.0.

| Data | | test | Sample 1 | n | Sample 2 | n | p-value | call | KS normality test |
|---|---|---|---|---|---|---|---|---|---|
| Figure 2G | | Student t-test | vehicle | 5 | T-025 50 mg/kg | 5 | < 0.0001 | *** | yes |
| Figure 2H | | Student t-test | vehicle | 5 | T-025 50 mg/kg | 5 | 0.433 | n.s. | yes |
| Figure 3B | | Mann-Whitney | MYC Amp | 29 | MYC non Amp | 121 | 0.0042 | ** | no |
| Figure 3C | | Mann-Whitney | CLK2 high | 50 | CLK2 low | 50 | < 0.0001 | *** | no |
| Figure 4A | | Mann-Whitney | CLK2 protein high | 10 | CLK2 protein low | 10 | 0.0001 | *** | no |
| Figure 5A | | t-test (Welch's correction) | SK-MEL-28 vector | 3 | SK-MEL-28 myc | 3 | 0.0003 | *** | yes |
| FIgure 5B | | Student t-test | SK-MEL-28 vector | 3 | SK-MEL-28 myc | 3 | < 0.0001 | *** | yes |
| FIgure 5F | | Student t-test | SK-MEL-28 vector | 3 | SK-MEL-28 myc | 3 | < 0.0001 | *** | yes |
| Figure 6A | CLK1 | Student t-test | SK-MEL-28 vector | 3 | SK-MEL-28 myc | 3 | 0.269 | n.s. | yes |
| | CLK2 | Student t-test | SK-MEL-28 vector | 3 | SK-MEL-28 myc | 3 | 0.841 | n.s. | yes |
| | CLK3 | Student t-test | SK-MEL-28 vector | 3 | SK-MEL-28 myc | 3 | 0.096 | n.s. | yes |
| | CLK4 | Student t-test | SK-MEL-28 vector | 3 | SK-MEL-28 myc | 3 | 0.589 | n.s. | yes |
| | SRSF1 | Student t-test | SK-MEL-28 vector | 3 | SK-MEL-28 myc | 3 | 0.0354 | * | yes |
| | PRMT5 | Student t-test | SK-MEL-28 vector | 3 | SK-MEL-28 myc | 3 | 0.0057 | ** | yes |
| Figure 7A | | t-test (Welch's correction) | vehicle | 8 | T-025 50 mg/kg | 8 | 0.0003 | *** | yes |
| Figure 7B | | Student t-test | vehicle | 8 | T-025 50 mg/kg | 8 | 0.0004 | *** | yes |
| Figure EV2B | | Mann-Whitney | MYC altered | 10 | MYC non altered | 9 | 0.905 | n.s. | no |
| Figure EV2C | | Mann-Whitney | MYC family altered | 12 | MYC family non altered | 7 | 0.800 | n.s. | no |
| Figure EV2D | | Mann-Whitney | MYC family altered | 37 | MYC family non altered | 113 | 0.001 | *** | no |
| Figure EV2E | | Mann-Whitney | CLK2 high | 6 | CLK2 low | 6 | 1 | n.s. | no |
| Figure EV3A | | Mann-Whitney | vehicle | 5 | T-025 50 mg/kg | 5 | 0.0079 | ** | no |
| Figure EV3B | | t-test (Welch's correction) | vehicle | 5 | T-025 50 mg/kg | 5 | 0.339 | n.s. | yes |
| Figure EV4E | | Mann-Whitney | CLK2 protein high | 3 | others | 5 | 0.0357 | * | no |
| Figure EV5B | Breast cancer | Mann-Whitney | MYC-Amp/CLK2 high | 5 | Ohters | 11 | 0.0174 | * | no |
| | | Mann-Whitney | MYC-Amp | 7 | MYC non Amp | 9 | 0.0712 | n.s | no |
| | Lung cancer | Mann-Whitney | MYC family Amp/CLK2 high | 5 | Ohters | 13 | 0.767 | n.s | no |
| | | Mann-Whitney | MYC family Amp | 8 | MYC family non Amp | 10 | 0.122 | n.s | no |
| | Colon cancer | Mann-Whitney | MYC-Amp/CLK2 high | 3 | Ohters | 14 | 0.147 | n.s | no |
| | | Mann-Whitney | MYC-Amp | 6 | MYC non Amp | 11 | 0.0182 | * | no |
| | CNS cancer | Mann-Whitney | MYC family Amp/CLK2 high | 3 | Ohters | 12 | 0.097 | n.s | no |
| Appendix Fig S5A | | Student t-test | MYC-Amp | 29 | MYC non-Amp | 121 | 0.0026 | ** | yes |
| Appendix Fig S6A | | Mann-Whitney | MYC Amp | 492 | MYC non Amp | 1374 | 0.248 | n.s. | no |

Statistical analysis below were performed by using EXSUS version 8.0.

| Data | | test | Sample 1 | n | Sample 2 | n | p-value | call | KS normality test |
|---|---|---|---|---|---|---|---|---|---|
| Figure 4E | | Steel-Dwass test | NCI-H1048 100 nmol/L | 125 | MDA-MB-468 100nmol/L | 125 | < 0.0001 | *** | no |
| | | | MDA-MB-468 100nmol/L | 125 | COLO320HSR 100nmol/L | 125 | 0.0129 | * | |
| | | | COLO320HSR 100nmol/L | 125 | 786-O 100nmol/L | 125 | < 0.0001 | *** | |
| Figure EV3D | at Day 15 | Dunnett's test | vehicle | 5 | T-025 37.5 mg/kg | 5 | < 0.0001 | *** | yes |
| | | | vehicle | 5 | T-025 50 mg/kg | 5 | < 0.0001 | *** | |
| Figure EV3E | at Day 15 | Dunnett's test | vehicle | 3 | T-025 25 mg/kg | 3 | 0.0298 | * | yes |
| | | | vehicle | 3 | T-025 50 mg/kg | 3 | 0.0037 | ** | |
| Figure 5D | 125 nmol/L | Tukey's Test | vector | 3 | vector + Dox | 3 | 0.648 | n.s. | yes |
| | | | myc | 3 | myc + Dox | 3 | 0.0001 | *** | |
| | | | vector | 3 | myc | 3 | 0.999 | n.s. | |
| Figure 6F | | Tukey's Test | Control | 3 | Dox | 3 | 0.0789 | n.s. | yes |
| | | | Control | 3 | T-025 | 3 | < 0.0001 | *** | |
| | | | Dox | 3 | Dox + T-025 | 3 | < 0.0001 | *** | |
| | | | T-025 | 3 | Dox + T-025 | 3 | 0.998 | n.s. | |
| Figure 6G | | Tukey's Test | Control | 3 | Dox | 3 | < 0.0001 | *** | yes |
| | | | Dox | 3 | Dox + T-025 | 3 | < 0.0001 | *** | |
| Appdendix Fig S4B | MCF7 DYRK1A | Tukey's Test | NS_1 | 3 | siDRYK1A_1 | 3 | < 0.0001 | *** | yes |
| | | | NS_1 | 3 | siDYRK1A_2 | 3 | < 0.0001 | *** | |
| | | | NS_2 | 3 | siDRYK1A_1 | 3 | < 0.0001 | *** | |
| | | | NS_2 | 3 | siDYRK1A_2 | 3 | < 0.0001 | *** | |
| | SKBR3 DYRK1A | Tukey's Test | NS_1 | 3 | siDRYK1A_1 | 3 | < 0.0001 | *** | yes |
| | | | NS_1 | 3 | siDYRK1A_2 | 3 | < 0.0001 | *** | |
| | | | NS_2 | 3 | siDRYK1A_1 | 3 | 0.0001 | *** | |
| | | | NS_2 | 3 | siDYRK1A_2 | 3 | < 0.0001 | *** | |
| Appendix Fig S5C | SK-MEL-28 vector | Tukey's Test | Control | 3 | T-025 | 3 | < 0.0001 | *** | yes |
| | | | Dox | 3 | Dox + T-025 | 3 | < 0.0001 | *** | |
| | SK-MEL-28 myc | Tukey's Test | Control | 3 | T-025 | 3 | < 0.0001 | *** | yes |
| | | | Dox | 3 | Dox + T-025 | 3 | 0.0004 | *** | |

**Appendix Figure S9.** Detailed result of statistical test

Detailed results of statistical test in each figure are shown. All tests were performed by using Prism 5.0 or EXSUS version 8.0.

## Appendix Supplemental Methods

The enzymatic assays (Fig EV4) were performed as previously described (Funnell et al, 2017).

## Cell lines

The origin and culture medium of cell lines used in Appendix Fig S3 were shown below.

| Cell line | Vender | Medium |
|---|---|---|
| 5637 | RIKEN | RPMI1640 + 10% FBS |
| 143B | ATCC | RPMI1640 + 10% FBS |
| A2780 | DS Pharmabiomedical | RPMI1640 + 10% FBS |
| A549 | ATCC | RPMI1640 + 10% FBS |
| BxPC-3 | ATCC | RPMI1640 + 10% FBS |
| Caki-2 | ATCC | RPMI1640 + 10% FBS |
| Caov-3 | ATCC | DMEM + 10% FBS |
| Caov-4 | ATCC | L-15 + 20% FBS |
| CCRF-CEM | ATCC | RPMI1640 + 10% FBS |
| Colo320 DM | JCRB | DMEM + 10% FBS |
| DLD-1 | ATCC | RPMI1640 + 10% FBS |
| DU 145 | ATCC | DMEM + 10% FBS |
| G401 | ATCC | McCOY's 5A + 10% FBS |
| G402 | ATCC | RPMI1640 + 10% FBS |
| H4 | ATCC | DMEM + 10% FBS |
| HCT116 | ATCC | McCOY's 5A + 10% FBS |
| HCT15 | ATCC | RPMI1640 + 10% FBS |
| HH | ATCC | RPMI1640 + 10% FBS |
| HL-60 | ATCC | RPMI1640 + 10% FBS |
| HNT34 | RIKEN | RPMI1640 + 10% FBS |
| HPAF-II | ATCC | E-MEM + 10% FBS + Sodium Pyruvate + NEAA |

| | | |
|---|---|---|
| HPB-ALL | DSMZ | RPMI1640 + 20% FBS |
| Hs746T | ATCC | DMEM + 10% FBS |
| HT-29 | ATCC | McCOY's 5A + 10% FBS |
| KO52 | JCRB | MEM alpha + 10% FBS |
| KP-3 | JCRB | RPMI1640 + 10% FBS |
| KYSE70 | JCRB | RPMI1640 + 10% FBS |
| LS174T | ATCC | E-MEM + 10% FBS + Sodium Pyruvate + NEAA |
| MIA-PaCa-2 | ATCC | DMEM +10% FBS +2.5% Horse serum |
| NCI-H2106 | ATCC | DMEM/F12 + 5% FBS + 0.005 mg/ml Insulin + 0.01 mg/ml Transferrin +30nM Sodium selenite +10 nM Hydrocortisone +10 nM beta-estradiol +extra 2mM L-glutamine |
| NCI-H460 | ATCC | RPMI1640 + 10%FBS + Sodium Pyruvate |
| NCI-H661 | ATCC | RPMI1640 + 10% FBS |
| NKM-1 | JCRB | RPMI1640 + 10% FBS |
| PA-1 | ATCC | E-MEM + 10% FBS + Sodium Pyruvate + NEAA |
| Panc1 | DS Pharmabiomedical | RPMI1640 + 10% FBS |
| PC3 | ATCC | RPMI1640 + 10% FBS |
| RCC4, vector | DS Pharmabiomedical | RPMI1640 + 10% FBS |
| RCC4, VHL | DS Pharmabiomedical | RPMI1640 + 10% FBS |
| Reh | ATCC | RPMI1640 + 10% FBS + Sodium Pyruvate |
| RKO | ATCC | E-MEM + 10% FBS + Sodium Pyruvate + NEAA |
| RS4;11 | ATCC | RPMI1640 + 10% FBS |
| SKOV3 | ATCC | McCOY's 5A + 10% FBS |
| SU.86.86 | ATCC | RPMI1640 + 10% FBS |
| SUP-B15 | ATCC | IMDM + 20% FBS + 0.05 mM Monothioglycelol Solution |
| SUP-T1 | ATCC | RPMI1640 + 10% FBS |
| SW1088 | ATCC | L-15 + 10% FBS |
| SW480 | ATCC | L-15 + 10% FBS |
| SW948 | ATCC | L-15 + 10% FBS |
| T-24 | ATCC | RPMI1640 + 10% FBS |
| THP-1 | ATCC | RPMI1640 + 20% FBS + 0.05mM 3-mercapto-1,2-propandiol |
| TMD5 | JCRB | MEM Alpha + 10%FBS |

## Primers, probes, and standard oligonucleotides

The following primers, probes, and standard oligonucleotide were used for *BCLAF1*:

*BCLAF1* (Ex10–11)_F, 5′-CAG GAG TTA GCC GAC CAC G-3′

*BCLAF1* (Ex10–11)_FAM, 5′-AAC CTT TTT TCG AAT TAG AGG CA-3′

*BCLAF1* (Ex10–11)_R, 5′-GTT TGG ACC AGT ATT TGT CCC AG-3′

*BCLAF1* (Ex10–12)_F, 5′-TGC AGG AGT TAG CCG ACC AC-3′

*BCLAF1* (Ex10–12)_FAM, 5′-AAC CTT TCA TGA CGA CAG AG-3′

*BCLAF1* (Ex10–12)_R, 5′-TTG GCC CAA TAA TCC ACA CC-3′

Standard oligo-nucleotides:

*BCLAF1* (Ex10–11), 5′-CTT TGC AGG AGT TAG CCG ACC ACG AGG AAC CTT TTT TCG AAT TAG AGG CAG AGG AAG AGC CAG AGG AGT TTT TGC TGG GAC AAA TAC TGG TCC AAA C-3′

*BCLAF1* (Ex10–12), 5′-AGG CTT TGC AGG AGT TAG CCG ACC ACG AGG AAC CTT TCA TGA CGA CAG AGA TGA TGG TGT GGA TTA TTG GGC CAA AAG AGG-3′.

The following primers, probes, and standard oligonucleotide were used for *NOP16*:

*NOP16* (Ex2–3)_F, 5′-CGC TAA ATC GGT ACG GCA GA-3′

*NOP16* (Ex2–3)_FAM, 5′-TAA GAG AAA GGT GAA GGC C-3′

*NOP16* (Ex2–3)_R, 5′-GCA CAT AGG GCT TCC GTA CAA G-3′

*NOP16* (Ex2–4)_F, 5′-ACC ACG CTA AAT CGG TAC GG-3′

*NOP16* (Ex2–4)_FAM, 5′-AAA GAC CTG GAG GCA GAA-3′

*NOP16* (Ex2–4)_R, 5′-GTC AAT GAG GTC CCG AGA CAG-3′

Standard oligo-nucleotides:

*NOP16* (Ex2–3), 5′-GAC CAC GCT AAA TCG GTA CGG CAG AAC CTG GCC GAG ATG GGG TTG GCT GTG GAC CCC AAC AGG GCG GTG CCC CTC CGT AAG AGA AAG GTG AAG GCC ATG GAG GTG GAC ATA GAG GAG AGG CCT AAA GAG CTT GTA CGG AAG CCC TAT GTG CTG AAT-3′.

*NOP16* (Ex2–4), 5′-CTG GGA CCA CGC TAA ATC GGT ACG GCA GAA CCT GGC CGA GAT GGG GTT GGC TGT GGA CCC CAA CAG GGC GGT GCC CCT CCG TAA GAG AAA GAC CTG GAG GCA GAA GCC AGC CTT CCA GAA AAG AAA GGA AAT ACT CTG TCT CGG GAC CTC ATT GAC TAT GT-3′

The following FAM-labeled probe and primer mix purchased from Thermo Fisher Scientific (Carlsbad, CA, U.S.A) were used:

CLK1 Hu00269734_m1

CLK2 Hs00241874_m1

CLK3 Hs00357427_m1

CLK4 Hu00982806_m1

SRSF1 Hs00199471_m1

PRMT5 Hs01047356_ml

MYC Hs00153408_ml

DYRK1A Hs00176369_m1

**Information of siRNAs**

Silencer Select Pre-designed siRNAs purchased from Ambion (Thermo Fisher Scientific) were used. The siRNA ID# and sequence are shown below:

siMYC_1 (s9129) 5′-AGA CCU UCA UCA AAA ACA Utt-3′

siMYC_2 (s9130) 5′-GAG CUA AAA CGG AGC UUU Utt-3′

siDYRK1A_1 (s4400) 5′-CCG UAA ACU UCA UAA CAU Utt-3′

siDYRK1A_2 (s4401) 5′-GCU GAC UAC UUG AAG UUC Att-3′

**Cell proliferation assay, Caspase-3/7 assay**

Cell proliferation was measured using CellTiter-Glo luminescent cell viability assay (Promega, Fitchburg, WI, U.S.A.). Caspase-3/7 activity was measured using Caspase-Glo 3/7 Assay Systems (Promega). Luminescence of each well was measured using ARVO Light (PerkinElmer Inc., Waltham, MA, U.S.A.). The $IC_{50}$ value and 95%

confidential intervals were estimated using GraphPad Prism 5 (GraphPad Software, Inc., La Jolla, CA, U.S.A.).

**Immunoblotting**

The same amount of proteins was electrophoresed on 7.5%–15% Perfect NT Gel (DRC CO ltd., Osaka, Japan) with sodium dodecyl sulfate (SDS) running buffer (Bio-Rad) at constant voltage. Gels were then transferred to nitrocellulose membrane using iBlot system (Invitrogen) at 20 V for 7 min, followed by 1 h incubation of the transferred membrane with PBS containing Block-Ace (DS Pharma Biomedical Co., Ltd., Osaka, Japan). Primary antibodies were diluted in Can Get Signal Immunoreaction Enhancer solution 1 (TOYOBO, Osaka, Japan), and the membranes were probed with the primary antibodies overnight at approximately 4C. After washing with PBST for 10 min, three times, the membranes were incubated with the secondary antibody for 30 min at room temperature (approximately 25°C), diluted at 1:10000, in Can Get Signal Immunoreaction Enhancer solution 2 (TOYOBO). After washing with PBST for 10 min, three times, the signals were detected using the ECL™ Western blotting analysis system (GE Healthcare, Waukesha, IL, U.S.A). All blots were imaged using an LAS3000 Luminescent Image Analyzer (Fujifilm, Tokyo, Japan) and quantified using Multi

Gauge V3.1 (Fujifilm).

**Immunohistochemistry**

Sections (5 μm) were cut and stained using Leica Bond Rx (Leica Biosystems, Wetzlar, Germany). Slides were pretreated with H2 buffer for 20 min and blocked with hydrogen peroxide for 5 min and protein block for 10 min (PVDF Blocking Reagent) followed by a 30-min incubation with the anti-pS98 CLK2 mAb (1:100 dilution) and detection using the Bond Polymer system/Leica IHC Refine Kit (DS9800, 10 min). pCLK2 staining was visualized using DAB. Sections were counterstained with hematoxylin.

**Protein expression and purification**

The cDNA encoding amino acid residues 130–495 of human CLK2 (GenBank NM_003993, Open Biosystems, currently GE Healthcare Dharmacon Inc., CO, U.S.) was cloned into a modified pET28a+ vector and engineered with an N-terminal hexahistidine tag linked by a TEV protease-cleavage site. The plasmid also contained Lambda phosphatase, which was co-expressed during the induction of CLK2. The plasmid was transformed into BL21(DE3) *E. coli*. Cells were grown at 37°C and

lowered to 18°C for 1 hour prior to overnight induction with 0.25 mmol/L IPTG. Cells were isolated by brief centrifugation and pellets were frozen and stored at −80°C. The cell pellet was lysed in buffer (50 mmol/L HEPES pH 7.6, 5% glycerol, 500 mmol/L NaCl, 5 mmol/L imidazole (buffer components were routinely purchased from Sigma-Aldrich), 0.5 mmol/L tris(2-carboxyethyl)phosphine (TCEP) (Thermo Fisher Scientific Co., MA, U.S.), and EDTA-free protease inhibitors (Roche, Basel, Switzerland)) by brief sonication (Sonics Vibra-Cell) and using a microfluidizer (Microfluidics M-110P).

The lysate was clarified by centrifugation and the supernatant was affinity purified using nickel-chelating resin (Life Technologies, currently Thermo Fisher Scientific Co.), followed by elution at room temperature with buffer composed of 50 mmol/L HEPES pH 7.6, 5% glycerol, 500 mmol/L NaCl, 300 mmol/L imidazole, and 0.5 mmol/L TCEP. As the CLK2 protein tended to precipitate, the solution was immediately flash frozen with liquid nitrogen in 1 mL aliquots at protein concentrations of 1–5 mg/mL for storage at −80°C. Protein concentration was estimated using a NanoDrop instrument.

**Crystallography**

Prior to crystallization, protein aliquots were quickly thawed at room temperature,

and T-025 was added to a final concentration of 0.1 mmol/L. The complex was co-concentrated using an Amicon concentrator (EMD Millipore, MA, U.S.) at 30°C to 10 mg/mL final protein concentration.

Crystallization was performed using the hanging drop vapor diffusion method with the reservoir solution: 0.1 mol/L HEPES (pH 7.0), 1–4% PEG MME 2000 (Hampton Research), 0.2 mol/L KBr, 0.2 KSCN, and 0.4–1.8% poly-γ-glutamic acid (Molecular Dimensions). Crystals were set up at room temperature, transferred to 4°C for growth (reaching maximum size within one week), cryo-protected with reservoir solution containing 30% ethylene glycol, and then flash-cooled in liquid nitrogen.

Diffraction data were collected at the Advanced Light Source (ALS) beamline 5.0.3 (Lawrence Berkeley National Laboratory) and processed using HKL2000 (Otwinowski & Minor, 1997). The structure was solved by molecular replacement with Phaser (McCoy et al, 2007) using the coordinates of human CLK2 (PDB code: 3NR9) as a search model. The graphics program COOT (Emsley & Cowtan, 2004) was used for model building, and refinement was performed with REFMAC5 (Murshudov et al, 1997). Phaser and REFMAC5 are distributed as part of CCP4 (1994). Structure validation was performed using Molprobity. Refinement statistics and an image of the omit electron density for the ligand are shown in Figure S3. The coordinates of the

structure were deposited in PDB under the accession code 5UNP.

**Auto-phosphorylation reaction of hCLK2 using γ($^{18}O_4$)-ATP**

Human recombinant CLK2 protein was dissolved in the reaction buffer (50 mmol/L Tris- HCl (pH7.5), 1 mmol/L $MgCl_2$, 1 mmol/L dithiothreitol (DTT), and 1 mmol/L γ($^{18}O_4$)-ATP) and the mixed solution was incubated for 2 hours at 37°C. The reaction was stopped by adding Laemmli sample buffer, subjected to SDS-PAGE, and stained using Coomassie brilliant blue. The CLK2 band at 85 kDa was excised. The excised gel was destained using 50 mmol/L triethylammonium bicarbonate (TEAB) in 50% ACN. A reduction step was performed by addition of 50 mmol/L TEAB/10 mmol/L DTT for 1 hour at room temperature. The proteins were alkylated by adding 50 mmol/L TEAB/40 mmol/L iodoacetamide and allowed to stand in the dark for 30 minutes at room temperature. The gel sections were alternately washed with 50 mmol/L TAEB and ACN. Digestion was carried out using 5 μg/mL sequencing grade modified trypsin in 50 mmol/L TEAB. Sufficient trypsin solution was added to swell the gel pieces followed by incubation at 37°C overnight. Peptides were extracted from the gel pieces with 30% ACN/0.1% TFA and 60% ACN/0.1% TFA and analyzed by LC-MS/MS.

**CLK2 purification and digestion**

293T cells transfected with FLAG-tagged CLK2 were lysed with PIPA buffer (50 mmol/L Tris-HCl (pH 7.5), 150 mmol/L NaCl, 2 mmol/L EDTA, and 1% NP-40 containing protein phosphatase inhibitor cocktail 1 and 2 (Sigma-Aldrich, currently EMD Millipore, MA, U.S.) and protease inhibitors (Sigma-Aldrich). FLAG-tagged CLK2 was purified by M2-agarose affinity beads (Sigma-Aldrich) according to the manufacturer's protocol. Purified CLK2 was subjected to SDS-PAGE and stained using Sypro Ruby (Bio Rad, CA, U.S.). The CLK2 band was excised and digested in-gel in the same manner as described above.

**LC-MS/MS analysis**

The extracted peptides were dissolved in 0.1% TFA with 2% ACN and analyzed by on-line nano LC using an Easy nLC1000 System coupled to Fusion Orbitrap mass spectrometer. The peptides were loaded onto a trap column (C18 Pepmap100, 3 μm, 0.075 × 20 mm) and resolved on an analytical column (Reprosil-Pur C18AQ 3 μm 0.075 × 150 mm) at 300 nL/minute over 45 minutes. The mass spectrometer was operated in a top-10 mode with dynamic exclusion of 10 seconds, collecting MS spectra

in the Orbitrap mass analyzer at a resolution of 120,000 and data-dependent HCD MS/MS spectra in the ion trap with normalized collision energy of 30%.

**Database search**

Peak list extractions from Xcalibur raw files were automatically performed using Proteome Discoverer 1.4 software (Thermo Fisher Scientific). Peak lists were searched against the Uniplot human protein database using Mascot software (version 2.5, Matrix Science). The mass tolerance of precursor and fragment were set to 10 ppm and 0.45 Da. Up to two missed trypsin cleavages were allowed.

For Mascot search, the following parameters were set: carbamidomethylation of cysteine was selected as a fixed modification; oxidation of methionine, phosphorylation of serine, threonine, and tyrosine were selected as variable modifications. Peptide identification data were routinely filtered to 1% false-discovery rate using the target-decoy strategy.

**Anti-pS98 CLK2 antibody generation**

A fifteen amino acid-peptide corresponding to Asp94-Tyr108 of human CLK2 (DYRH(pS)YEYQRENSSY) in which the Ser98 was phosphorylated was synthesized

with an added cysteine at the C-terminus by Scrum Inc. (Tokyo, Japan). The peptide was conjugated to Inject™ Maleimide-Activated mcKLH via the C-terminal cysteine as described above.

The same phosphorylated and non-phosphorylated peptides with C-terminal biotin (DYRH(pS)YEYQRENSSY-Bio, DYRHSYEYQRENSSY-Bio) were also synthesized by Scrum Inc. and were termed pCLK2 and CLK2 peptide, respectively.

Rabbits (Japanese White) were intradermally immunized with the KLH-conjugated peptide (250 μg) with Freund's Complete Adjuvant, followed by repeated immunization with the same amount of antigen with Freund's Incomplete Adjuvant every 2 weeks. After 56 days, the serum titers were evaluated with an ELISA using biotinylated CLK peptides, and splenocytes were collected from the rabbits demonstrating the optimal titer. Those experiments were performed at Mediridge Co. Inc.(Tokyo, Japan).

Rabbit monoclonal antibodies were generated from the splenocytes according to Kurosawa's method with modifications (Kurosawa et al, 2012). Briefly, the antigen specific B-cells were collected by FACS using the biotinylated phospho-peptide and a fluorescent labeled streptavidin, and cDNA of rabbit IgG was isolated from single antigen specific B-cells. The recombinant IgGs were transiently expressed in Expi293

cells and the culture supernatants were screened by an ELISA using biotinylated CLK2 peptide and pCLK2 peptide as described below. The phospho-peptide-specific clones were screened by western blotting using the lysate of HCT116 cells that had been treated or non-treated with a CLK2 inhibitor. The selected anti-pS98 CLK2 rabbit monoclonal antibody (clone CLK2-S98pRb1-01H03) was transiently expressed in Expi293 cells and purified from the culture supernatant by protein A affinity chromatography.

**ELISA for screening anti-phospho CLK2 rabbit monoclonal antibodies**

A 96-well ELISA plate was coated with NeutrAvidin, followed by blocking with Protein Free (PBS) Blocking Buffer. After washing the plate with PBS containing 0.05% Tween 20 (PBS-T), 10 nmol/L of four biotinylated peptides (CLK2 and pCLK2 peptides) were captured on the plate. After washing with PBS-T, anti-serum or culture supernatant of the hybridomas was added to the plate and incubated at room temperature for 1 hour. After washing with PBS-T, the bound antibody was detected with HRP-conjugated anti-rabbit IgG goat pAb, using TMB (3, 3', 5, 5'-tetramethylbenzidine) as a substrate. After stopping the enzyme reaction with 1N $H_2SO_4$, the absorbance at 450 nm was measured using a microplate reader.

**Panel of growth inhibition assay and subsequence bioinformatics analysis (at Eurofins Inc.)**

A growth inhibition assay panel and subsequent bioinformatics analysis were performed at Eurofins Inc. (MO, U.S.A.) by utilizing Oncopanel240 or 60.

According to the study reports from Eurofins Inc., cells were seeded into 384 well plates at a single cell density in standardized media. After 24 hours, the test compounds were added and the plate was incubated continuously for 72 hours. Cells were then fixed and stained to visualize nuclei. The number of nuclei was counted and normalized. Cellular response parameters were calculated using nonlinear regression to a sigmoidal single-site dose response model and the $IC_{50}$ value of each cell line was calculated.

Genomic features were binned into six distinct categories, each of which was considered to be a “biomarker-positive” grouping: Mutated (non-synonymous, coding, and non-coding mutations), Amplified, Deleted, Overexpressed, Mutated or amplified (gain-of-function), and Mutated or deleted (loss-of-function). Two orthogonal significance tests were performed on each genomic feature as follows.

Student’s t-test: For each genomic feature within each of the six above categories, $IC_{50}$ cell count values for biomarker-positive and biomarker-negative groups were

compared. Only biomarker-positive groups with a user-defined sample size were analyzed. Comparisons of cell count values were performed using an unpaired t-test assuming equal variance and were used to compute the significance ($p$-value) of each comparison.

Fisher's exact test: Top 25%, 50%, or lower 25% of cell lines were classified as "sensitive", "intermediate", or "resistant", respectively. The Fisher's exact test was performed to determine whether the observed frequency of a genomic feature in the sensitive or resistant groups was greater than expected by chance. "Intermediate" cell lines were omitted from the analysis.

A false-discovery rate-adjusted $p$-value ($q$-value) was computed with a null hypothesis of no difference between biomarker-positive and biomarker-negative groups. The $q$-value was calculated according the following formula:

$q = (p/\text{rank}) \times N$

where rank is the rank of the $p$-value and N is the number of conducted tests. The $q$-value calculation was performed within each of the six genomic feature categories.

**Synthesis of T-025**

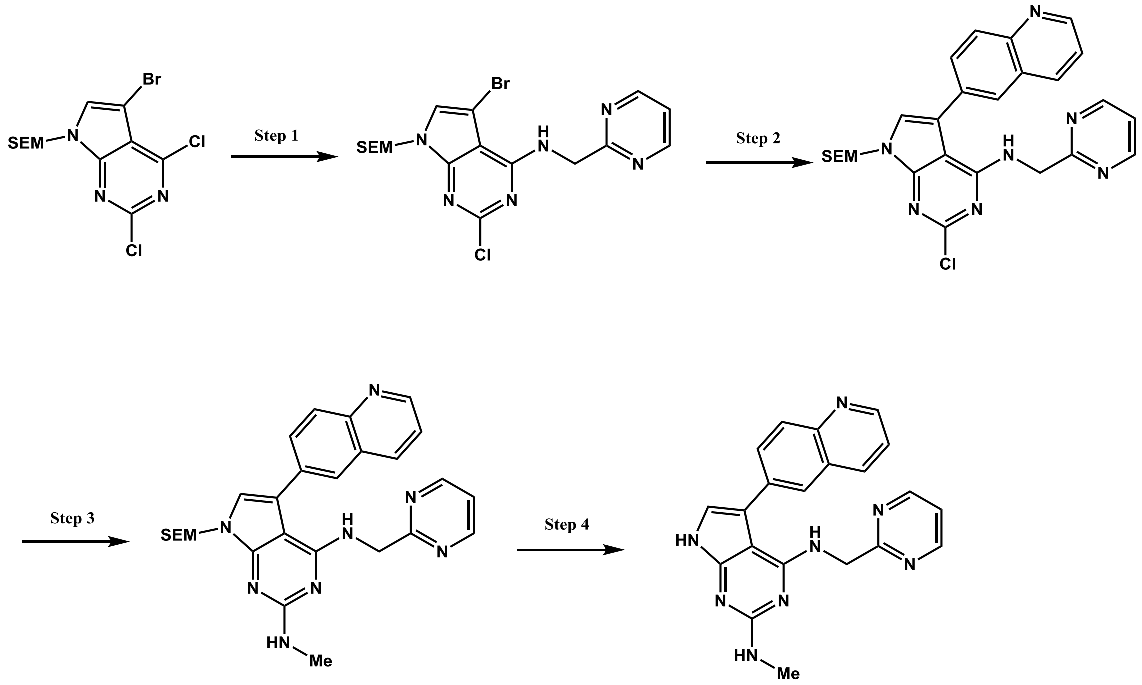


1)

**5-bromo-2-chloro-*N*-(pyrimidin-2-ylmethyl)-7-((2-(trimethylsilyl)ethoxy)methyl)-7*H*-pyrrolo[2,3-*d*]pyrimidin-4-amine**

To a mixture of 5-bromo-2,4-dichloro-7-((2-(trimethylsilyl)ethoxy)methyl)-7*H*-pyrrolo[2,3-*d*]pyrimidine (15 g, 37.77 mmol), pyrimidin-2-ylmethanamine hydrochloride (6.32 g, 43.43 mmol) and 2-propanol (150 mL) was added *N*-ethyl-*N*-isopropylpropan-2-amine (26.4 mL, 151.07 mmol) at room temperature. The mixture was stirred at 80 °C under $N_2$ overnight. The mixture was stirred at room temperature for 8h. The precipitation was collected by filtration and washed with IPE to give

5-bromo-2-chloro-*N*-(pyrimidin-2-ylmethyl)-7-((2-(trimethylsilyl)ethoxy)methyl)-7*H*-pyrrolo[2,3-*d*]pyrimidin-4-amine (16.0 g, 34.1 mmol, 90 %) as white solid.

$^1$H NMR (300 MHz, DMSO-$d_6$) δ -0.06 (9H, s), 0.76-0.91 (2H, m), 3.45-3.59 (2H, m), 4.91 (2H, d, J = 5.3 Hz), 5.43 (2H, s), 7.37-7.50 (1H, m), 7.64 (1H, s), 7.68-7.78 (1H, m), 8.82 (2H, d, J = 4.9 Hz).

2)

**2-chloro-*N*-(pyrimidin-2-ylmethyl)-5-(quinolin-6-yl)-7-((2-(trimethylsilyl)ethoxy)methyl)-7*H*-pyrrolo[2,3-*d*]pyrimidin-4-amine**

The mixture of 5-bromo-2-chloro-*N*-(pyrimidin-2-ylmethyl)-7-((2-(trimethylsilyl)ethoxy)methyl)-7*H*-pyrrolo[2,3-d]pyrimidin-4-amine (1 g, 2.13 mmol), quinolin-6-ylboronic acid (0.479 g, 2.77 mmol), potassium carbonate (0.882 g, 6.39 mmol) and TETRAKIS(TRIPHENYLPHOSPHINE)PALLADIUM(0) (0.123 g, 0.11 mmol) in DME (20 ml) and water (5 ml) was stirred at 80 °C overnight. After cooling to room temperature, the mixture was extracted with AcOEt and water . The organic layer was washed with brine, dried over $MgSO_4$ and concentrated *in vacuo*. The residue was purified by column chromatography (silica gel, eluent: AcOEt/Hexane = 0/100 to 100/0

and then 10% MeOH) to afford 2-chloro-*N*-(pyrimidin-2-ylmethyl)-5-(quinolin-6-yl)-7-((2-(trimethylsilyl)ethoxy)methyl)-7*H*-pyrrolo[2,3-*d*]pyrimidin-4-amine (220 mg, 0.425 mmol, 19.95 %) as pale yellow powder.

1H NMR (300 MHz, DMSO-$d_6$) δ ppm -0.04 (s, 9 H) 0.83 - 0.99 (m, 2 H) 3.54 - 3.70 (m, 2 H) 4.83 (d, J=4.71 Hz, 2 H) 5.55 (s, 2 H) 7.02 (t, J=4.90 Hz, 1 H) 7.37 (t, J=4.90 Hz, 1 H) 7.59 (dd, J=8.29, 4.14 Hz, 1 H) 7.68 (s, 1 H) 7.96 (dd, J=8.67, 2.07 Hz, 1 H) 8.09 - 8.23 (m, 2 H) 8.30 - 8.39 (m, 1 H) 8.55 (d, J=4.90 Hz, 2 H) 8.94 (dd, J=4.33, 1.70 Hz, 1 H).

3)

**$N^2$-methyl-$N^4$-(pyrimidin-2-ylmethyl)-5-(quinolin-6-yl)-7-((2-(trimethylsilyl)ethoxy)methyl)-7*H*-pyrrolo[2,3-*d*]pyrimidine-2,4-diamine**

The mixture of 2-chloro-*N*-(pyrimidin-2-ylmethyl)-5-(quinolin-6-yl)-7-((2-(trimethylsilyl)ethoxy)methyl)-7*H*-pyrrolo[2,3-*d*]pyrimidin-4-amine (1 g, 1.93 mmol), methanamine (2 mol/l in THF) (1.448 ml, 2.90 mmol), $Pd_2(dba)_3$ (0.177 g, 0.19 mmol), 2,2'-bis(diphenylphosphino)-1,1'-binaphthalene (0.240 g, 0.39 mmol), sodium

2-methylpropan-2-olate (0.556 g, 5.79 mmol) and 1,4-dioxane (9.0 ml) was heated at 100 °C for 3 h under microwave irradiation. The mixture was quenched with water and extracted with EtOAc. The organic layer was separated, washed with water and brine, dried over $Na_2SO_4$ and concentrated in vacuo. The residue was purified by column chromatography (amino silica gel, eluted with 63% - 93% EtOAc in hexane) to give

*N*$^2$-methyl-*N*$^4$-(pyrimidin-2-ylmethyl)-5-(quinolin-6-yl)-7-((2-(trimethylsilyl)ethoxy)methyl)-7*H*-pyrrolo[2,3-*d*]pyrimidine-2,4-diamine (930 mg, 1.814 mmol, 94 %) as pale orange solid. This product was subjected to the next reaction without further purification.

$^1$H NMR (300 MHz, DMSO-*d*$_6$) δ ppm -0.04 (s, 9 H) 0.79 - 0.97 (m, 2 H) 2.80 (d, *J*=4.90 Hz, 3 H) 3.48 - 3.69 (m, 2 H) 4.71 - 4.94 (m, 2 H) 5.45 (s, 2 H) 6.21 - 6.48 (m, 2 H) 7.14 - 7.21 (m, 1 H) 7.30 - 7.38 (m, 1 H) 7.52 - 7.60 (m, 1 H) 7.88 - 7.98 (m, 1 H) 8.05 - 8.16 (m, 2 H) 8.28 - 8.38 (m, 1 H) 8.54 (d, *J*=4.90 Hz, 2 H) 8.91 (dd, *J*=4.33, 1.70 Hz, 1 H).

4)

***N*$^2$-methyl-*N*$^4$-(pyrimidin-2-ylmethyl)-5-(quinolin-6-yl)-7*H*-pyrrolo[2,3-*d*]pyrimidin**

**e-2,4-diamine**

A solution of $N^2$-methyl-$N^4$-(pyrimidin-2-ylmethyl)-5-(quinolin-6-yl)-7-((2-(trimethylsilyl)ethoxy)methyl)-7*H*-pyrrolo[2,3-*d*]pyrimidine-2,4-diamine (930 mg, 1.81 mmol) in TFA (10mL, 129.80 mmol) was stirred at room temperature for 30 min. The mixture was concentrated azeotropic with toluene to give *N*-hydroxymethyl intermediate. To the residue, were added DMF(dry) (3 ml) and 2 mol/L $NH_3$-MeOH (7 mL). The mixture was stirred at 50 °C. for 30 min. The mixture was poured into iced water at room temperature and extracted with EtOAc-THF (X 2 times). The organic layer was separated, washed with brine , dried over $MgSO_4$ and concentrated *in vacuo*. The residue was purified by column chromatography (silica gel, eluted with 0% - 10% MeOH in EtOAc) to give pale green solid. The solid was crystallized from EtOH, EtOAc and IPE to give $N^2$-methyl-$N^4$-(pyrimidin-2-ylmethyl)-5-(quinolin-6-yl)-7*H*-pyrrolo[2,3-*d*]pyrimidine-2,4-diamine (190mg, 0.497 mmol, 27.4 %) as pale green solid.

$^1$H NMR (300 MHz, DMSO-$d_6$) δ ppm 2.77 (d, *J*=4.90 Hz, 3 H) 4.81 (d, *J*=4.71 Hz, 2 H) 6.04 - 6.20 (m, 1 H) 6.22 - 6.40 (m, 1 H) 7.00 (d, *J*=2.45 Hz, 1 H) 7.24 - 7.38 (m, 1 H) 7.50 - 7.69 (m, 1 H) 7.87 - 8.13 (m, 3 H) 8.25 - 8.36 (m, 1 H) 8.55 (d, *J*=4.90 Hz, 2

H) 8.81 - 8.96 (m, 1 H) 11.26 (br. s., 1 H). Anal. Calcd for $C_{21}H_{18}N_8$: C,65.95; H,4.74; N,29.30. Found: C,64.92; H,4.93; N,28.03.

**Appendix References**

(1994) The CCP4 suite: programs for protein crystallography. *Acta Crystallogr D Biol Crystallogr* **50:** 760-763

Emsley P, Cowtan K (2004) Coot: model-building tools for molecular graphics. *Acta Crystallogr D Biol Crystallogr* **60:** 2126-2132

Funnell T, Tasaki S, Oloumi A, Araki S, Kong E, Yap D, Nakayama Y, Hughes CS, Cheng SG, Tozaki H, Iwatani M, Sasaki S, Ohashi T, Miyazaki T, Morishita N, Morishita D, Ogasawara-Shimizu M, Ohori M, Nakao S, Karashima M et al (2017) CLK-dependent exon recognition and conjoined gene formation revealed with a novel small molecule inhibitor. *Nat Commun* **8:** 7

Kurosawa N, Yoshioka M, Fujimoto R, Yamagishi F, Isobe M (2012) Rapid production of antigen-specific monoclonal antibodies from a variety of animals. *BMC Biol* **10:** 80

McCoy AJ, Grosse-Kunstleve RW, Adams PD, Winn MD, Storoni LC, Read RJ (2007) Phaser crystallographic software. *J Appl Crystallogr* **40:** 658-674

Murshudov GN, Vagin AA, Dodson EJ (1997) Refinement of macromolecular structures by the maximum-likelihood method. *Acta Crystallogr D Biol Crystallogr* **53:** 240-255

Otwinowski Z, Minor W (1997) Processing of X-ray diffraction data collected in oscillation mode. *Methods Enzymol* **276:** 307-326
