# Supplementary material for: Anti‐tumor efficacy of a novel CLK inhibitor via targeting RNA splicing and MYC‐dependent vulnerability
Source: EMBO Mol Med. 2018 May 16;10(6):e8289. doi: 10.15252/emmm.201708289 (PMC5991599; doi:10.15252/emmm.201708289)
Supplement: Supplementary file 2 — Expanded View Figures PDF [file EMMM-10-e8289-s002.pdf]

Expanded View Figures

A

P-Value Rank	Gene	Probe	Fold Change	P-Value	Q-Value	Sensitive Mean	Sensitive StdDev	Resistant Mean	Resistant StdDev
1	WDR18	209461_x_at	-1.78	5.86E-13	2.51E-08	1149.9	387.3	646.7	262.9
2	RBMX_SNORD61	213762_x_at	-1.42	5.95E-13	1.28E-08	8631.4	1888.6	6083.9	1435.0
3	CD63	200663_at	1.61	6.22E-13	8.89E-09	7476.3	2994.3	12065.6	3062.3
4	CAD	202715_at	-1.78	7.39E-13	7.92E-09	2144.3	767.7	1202.0	432.1
5	SFXN4	229236_s_at	-1.88	1.22E-12	1.04E-08	742.8	239.6	395.3	226.0
6	SFXN4	225143_at	-1.86	2.67E-12	1.91E-08	1485.1	483.1	799.6	453.3
7	PDCD11	212422_at	-1.69	3.53E-12	2.16E-08	1092.7	373.0	647.7	218.2
8	GTPBP3	213835_x_at	-1.33	1.11E-11	5.94E-08	1391.3	284.4	1049.4	190.0
9	ANXA5	200782_at	1.97	2.35E-11	1.12E-07	4240.1	2296.0	8333.1	3505.5
10	APEX1	210027_s_at	-1.39	2.83E-11	1.21E-07	9272.5	2106.1	6649.9	1674.7
**70**	**CLK2**	**203229_s_at**	**-1.47**	**1.58E-09**	**9.66E-07**	**2207.8**	**670.0**	**1501.2**	**461.9**
	CLK1	214683_s_at	-1.21	3.69.E-02	1.35.E-01	561.8	275.9	466.2	203.5
	CLK3	238072_at	1.06	1.09.E-01	2.79.E-01	154.1	31.5	162.7	24.6
	CLK4	228751_at	1.11	2.34.E-01	4.47.E-01	117.8	43.4	130.2	66.2
	CLK4	241403_at	-1.06	2.54.E-01	4.69.E-01	63.2	19.0	59.4	16.3
	CLK3	202140_s_at	-1.05	3.38.E-01	5.53.E-01	1014.9	318.5	968.9	171.0
	CLK4	1568836_at	-1.03	6.00.E-01	7.65.E-01	40.5	10.8	39.4	11.3
	CLK4	210346_s_at	1.01	8.84.E-01	9.40.E-01	439.9	156.6	444.6	186.2
	DYRK1A	209033_s_at	-1.20	9.53.E-03	5.17.E-02	1970.1	747.7	1644.7	560.2
	DYRK1A	211079_s_at	-1.06	8.83.E-02	2.43.E-01	109.3	20.2	103.0	19.2
	DYRK1B	217270_s_at	-1.03	4.19.E-01	6.26.E-01	179.0	31.2	174.2	32.8
	DYRK1A	239308_at	1.02	4.78.E-01	6.74.E-01	137.5	19.1	140.4	24.2
	DYRK1A	211541_s_at	1.01	5.38.E-01	7.21.E-01	313.7	42.6	318.2	35.3
	DYRK1B	204954_s_at	1.01	8.57.E-01	9.27.E-01	214.6	38.6	216.2	56.6

B

Gene	Change	*p*-value Student's t-test	*p*-value Fisher's exact test	Q-value	Effect
ZFP106	Mut	1.11E-03	2.83E-03	5.93E-02	Sensitive
FBN3	Mut	1.11E-03	3.13E-03	5.92E-02	Sensitive
AUTS2	Mut	1.20E-03	8.34E-04	6.15E-02	Sensitive
PCM1	Mut	1.69E-03	8.34E-04	7.39E-02	Sensitive
CREBBP	Mut	4.99E-03	4.63E-03	1.25E-01	Sensitive
UBR4	Mut	5.24E-03	1.20E-03	1.29E-01	Sensitive
CHD7	Mut	5.61E-03	9.63E-03	1.36E-01	Sensitive
ROBO2	Mut	6.60E-03	7.69E-03	1.49E-01	Sensitive
**MYC**	**Mut or Amp**	**6.66E-03**	**8.32E-03**	**2.67E-01**	**Sensitive**
ARFGEF1	Mut	7.96E-03	9.83E-03	1.65E-01	Sensitive
ODZ2	Mut or Del	8.12E-03	4.86E-03	4.14E-01	Sensitive
LRRN3	Mut or Del	8.17E-03	1.28E-03	2.78E-01	Sensitive
ASXL1	Amp	9.39E-03	1.77E-03	3.42E-01	Resistant

Figure EV1. Result of unbiased bioinformatics analysis.

A Genes significantly associated with the sensitivity are shown ranked by their *P*-value. Also shown are the results of CLK and DYRK family genes.

B Representative gene alterations and their statistical powers. Correlation between T-025 sensitivity and gene alterations in each cell line. The *P*-values were determined using Student's *t*-test and Fisher's exact test.

© 2018 Takeda Pharmaceutical Company

A

240 cancer cell lines
↓
CCLE database
↓
matched
169 cell lines
(Solid 150, Blood 19)
↓
Analysis

B

Name	Disease	IC_{50} (nmol/L)	CLK2 Expression	MYC alteration	Other MYC family
MOLT16	Leukemia	20.6	Low	Translocation	
BV173	Leukemia	21.8	Low		
MV-4-11	Leukemia	30.1	High		
U266B1	Myeloma	35.1			Amp (MYCN)
HEL9217	Leukemia	38.3	High		
DOHH2	Lymphoma	39.5		Translocation	
DB	Lymphoma	43.3	Low	Amp	
DAUDI	Lymphoma	52.5	High	Translocation	
NALM6	Leukemia	52.6	High		
ST486	Lymphoma	57.1		Driver mut	
CMLT1	Leukemia	69.7		Amp	
RAJI	Lymphoma	84.7		Driver Mut, Translocation	
RPMI8226	Myeloma	99.3	High	Amp	
EM2	Leukemia	104	Low		
L428	Lymphoma	116	Low	Amp	
K562	Leukemia	129			
HT	Lymphoma	228			
THP1	Leukemia	228	High		Amp (MYCL1)
MEG01	Leukemia	305	Low	Amp	

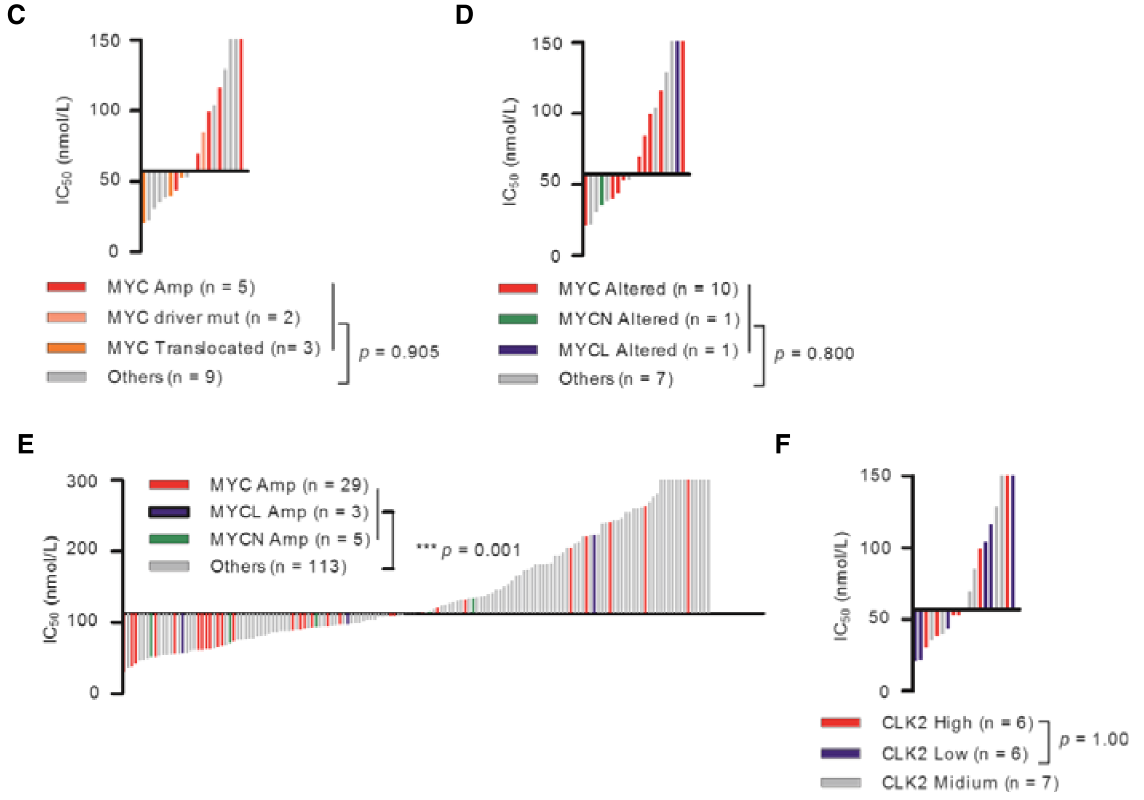

Figure EV2. Additional analysis of Oncopanel.

A Analysis flow of the tested cell lines.
B IC_{50} value, CLK2 expression, and *MYC* status of 19 hematological cancer cell lines are shown.
C Correlation between T-025 sensitivity and *MYC* status in the hematological cancer cell lines (n = 19). Each bar indicates a single cell line, and colored bar indicates cell lines with altered MYC.
D Correlation between T-025 sensitivity and MYC family gene status in the hematological cancer cell lines (n = 19). Each bar indicates a single cell line, and colored bar indicates cell lines with altered MYC family gene.
E Correlation between T-025 sensitivity and MYC family gene status in the solid cancer cell lines (n = 150). Each bar indicates a single cell line, and colored bar indicates cell lines with altered MYC family gene.
F Correlation between T-025 sensitivity and CLK2 expression in the hematological cancer cell lines (n = 19). Each bar indicates a single cell line, and blue, gray, or red bar indicates cell lines with high, medium, or low CLK2.

Data information: In (C–F), a Mann–Whitney test was performed.

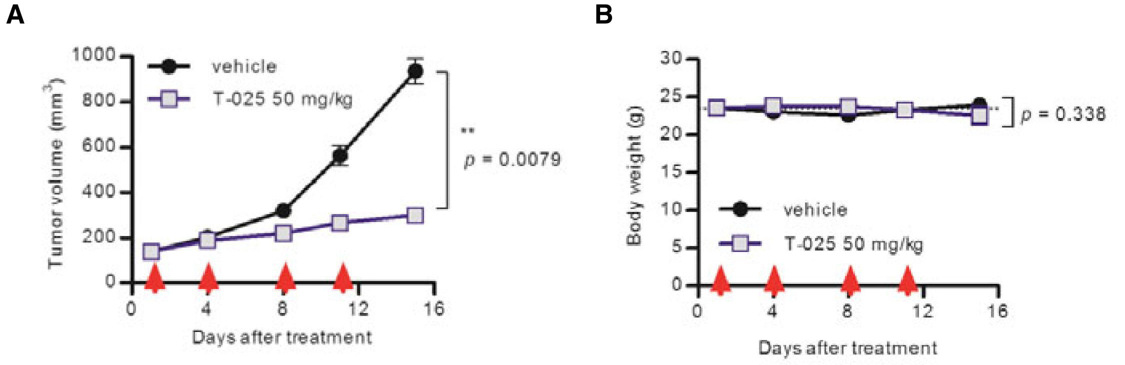

C

Name	Origin	IC_{50} (nmol/L) Cell count	Relative CLK2 expression
MV-4-11	Leukemia	30.1	0.6
CCRF-CEM	Leukemia	45.9	1.6
THP-1	Leukemia	228	0.9

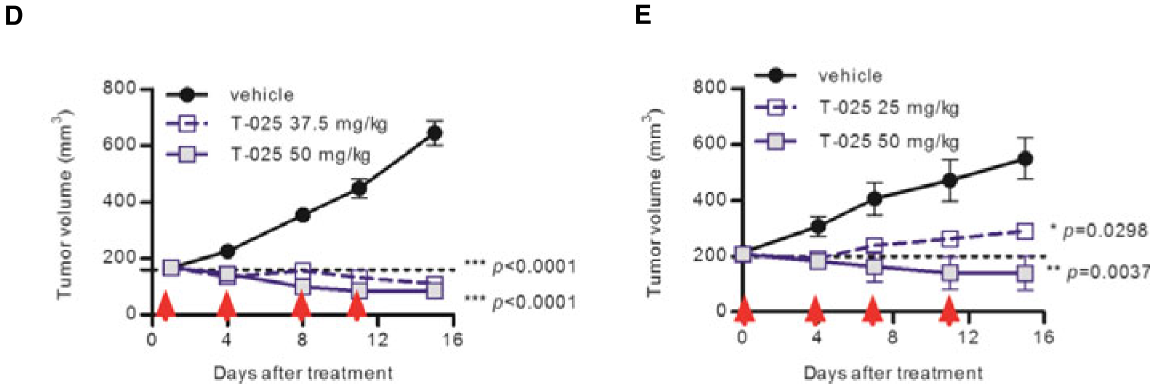

Figure EV3. Additional *in vivo* data of cell lines.

A, B Anti-tumor efficacy of T-025 in lung cancer NCI-H1048 xenograft model. Red arrows indicate the day of treatment in twice weekly regimens. Tumor volume (A) and body weight (B) during the treatment cycle are shown.

C Correlation between growth suppressive sensitivity to T-025 and the protein level of CLK2 in the hematological cancer cell lines.

D Anti-tumor efficacy of T-025 in acute myeloid leukemia MV-4-11 xenograft model.

E Anti-tumor efficacy of T-025 in patient-derived xenograft model of acute myeloid leukemia. T-025 was given twice daily at twice weekly.

Data information: In (A, B, and D), data are shown as the mean ± s.e.m. (n = 5). In (E), data are shown as the mean ± s.e.m. (n = 3). In (A), a Mann–Whitney test was performed. In (B), an unpaired Student's t-test was performed. In (D and E), Tukey's test was performed.

© 2018 Takeda Pharmaceutical Company

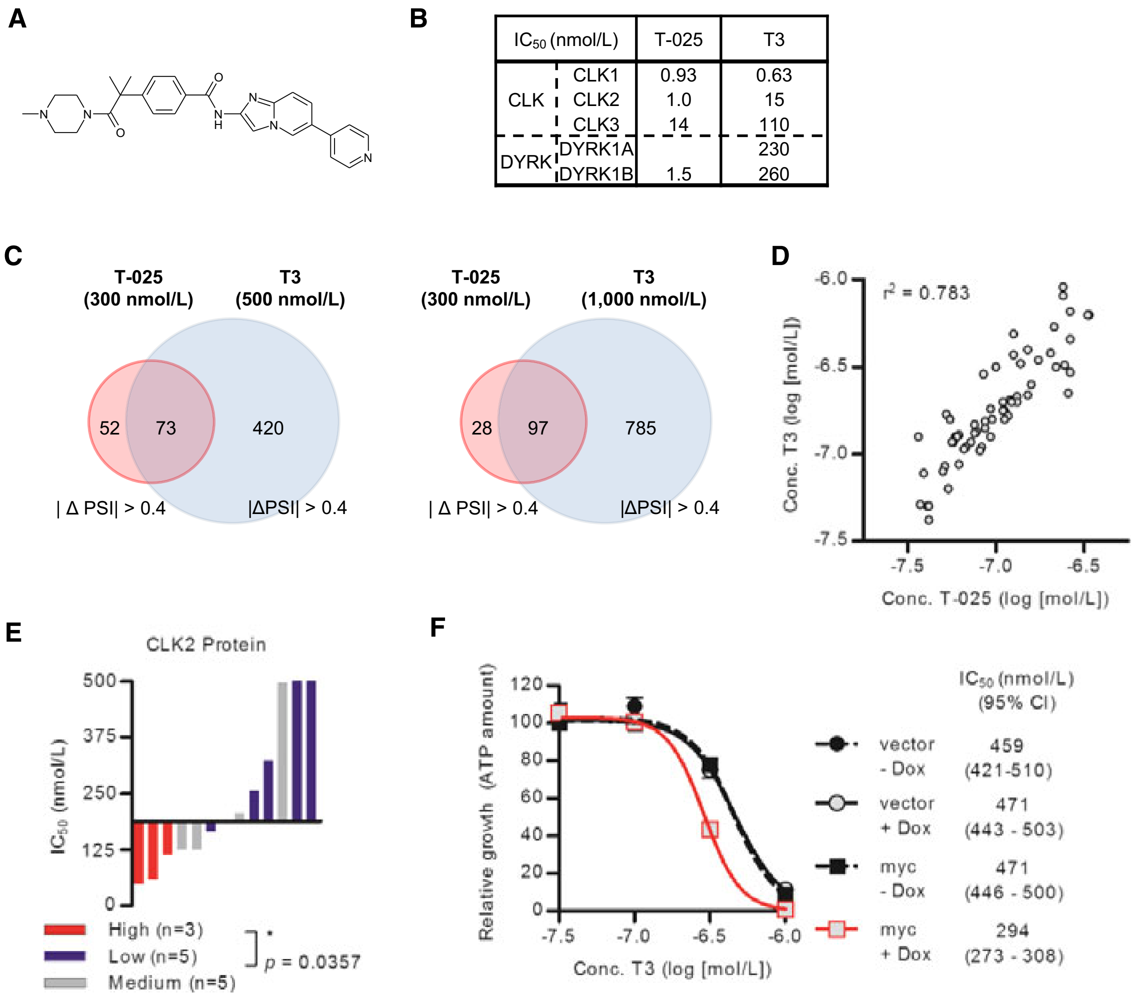

Figure EV4. The CLK inhibitors T3 and T-025 yielded similar profiles.

A Chemical structure of T3.

B The enzymatic assay results of T3 and T-025 against the CLK and DYRK family kinases.

C Comparison of SEs caused by T-025 and T3.

D Correlation between the IC_{50} values of T-025 and T3 in a panel of 60 cancer cell lines. Each circle indicates a single cell line. The r^2 value was calculated using Prism.

E Correlation between growth suppressive sensitivity to T3 and the protein level of CLK2 in cell lines in solid cancer cell lines. The definition of CLK2 protein expression was same as Fig 4A. A Mann–Whitney test was performed.

F Dose–response growth inhibition curve of T3 in MYC-inducible SK-MEL-28 cells. Cells pretreated with Dox for 48 h were additionally incubated with T-025 for 72 h. Data are shown as the means ± s.d. of three independent experiments (n = 3). IC_{50} values and 95% CI were determined by using Prism.

Data information: The chemical structure of T3, the result of enzymatic assay of T3, and AS events modulated by T3 in HCT116 are cited from a previous article (Funnell *et al*, 2017).

© 2018 Takeda Pharmaceutical Company

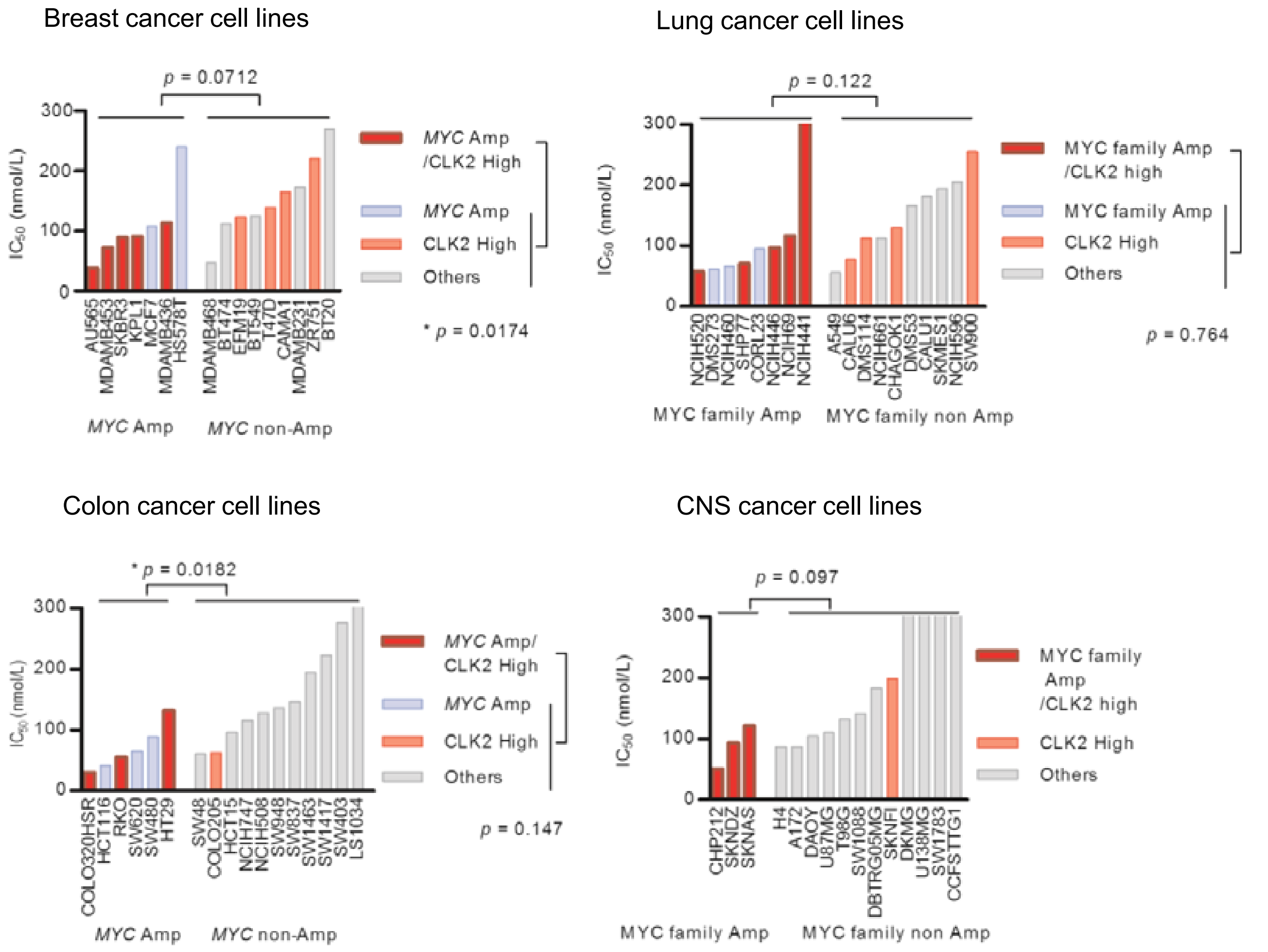

Figure EV5. Additional analysis of Oncopanel based on the original organ type.
IC_{50} values of the cancer cell lines with both high CLK2 and amplified *MYC* were compared with that without these biomarkers. A Mann–Whitney test was performed.

 © 2018 Takeda Pharmaceutical Company
