# Supplementary figures and images for: Anti‐tumor efficacy of a novel CLK inhibitor via targeting RNA splicing and MYC‐dependent vulnerability
Source: EMBO Mol Med. 2018 May 16;10(6):e8289. doi: 10.15252/emmm.201708289 (PMC5991599; doi:10.15252/emmm.201708289)

Fig 2A

pSR

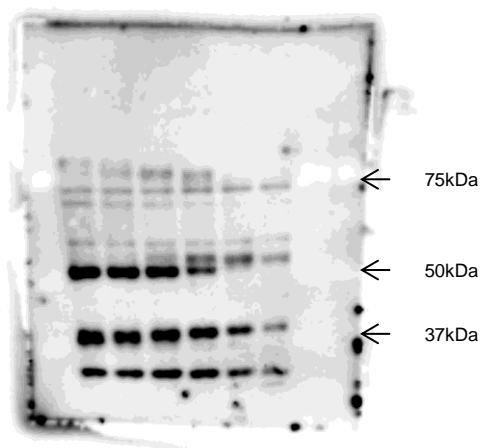

GAPDH

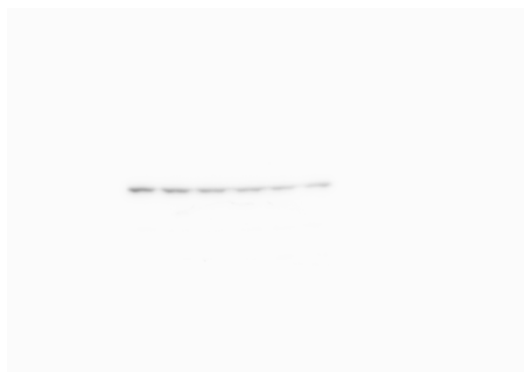

CLK2

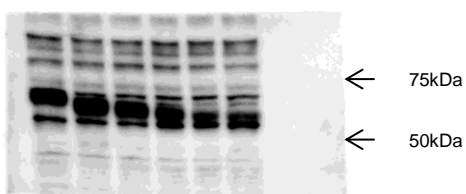

pCLK2

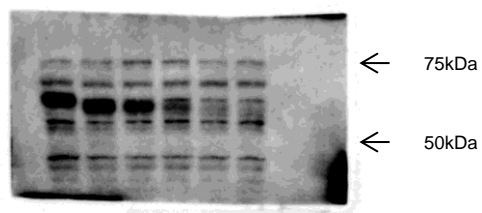

Fig 2E

pCLK2

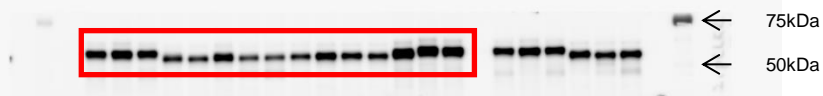

CLK2

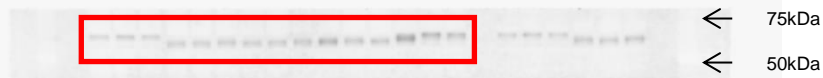

HSP90

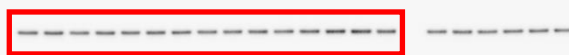

pSR

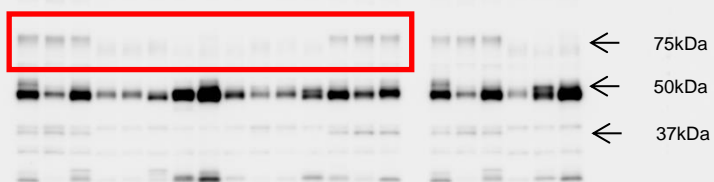

Supplement: Supplementary file 4 — Source Data for Figure 2 [file EMMM-10-e8289-s003.pdf]

Fig 4B

pCLK2

CLK2

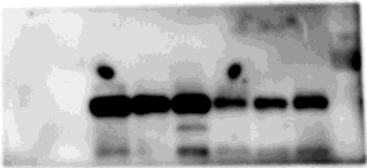

← 75kDa  
← 50kDa

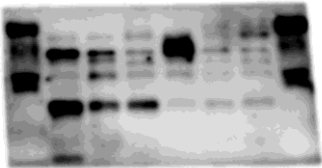

← 75kDa  
← 50kDa

GAPDH

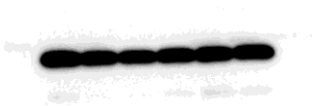

Supplement: Supplementary file 5 — Source Data for Figure 4 [file EMMM-10-e8289-s004.pdf]

Fig 5E

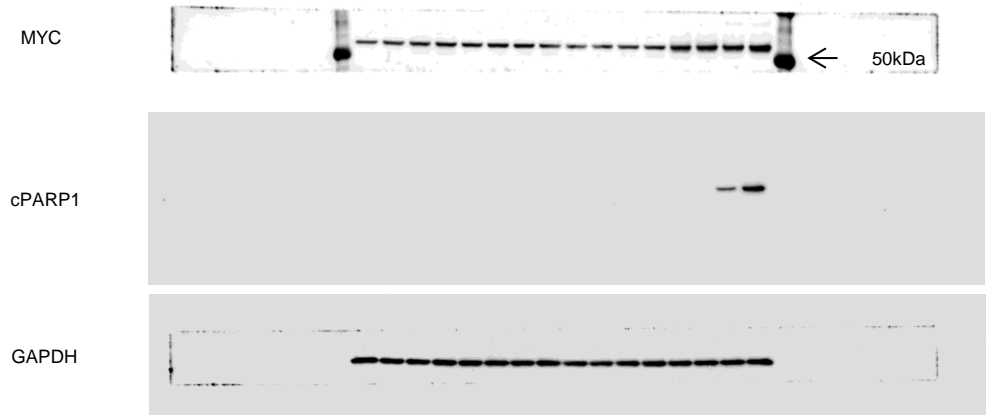

Fig 5H

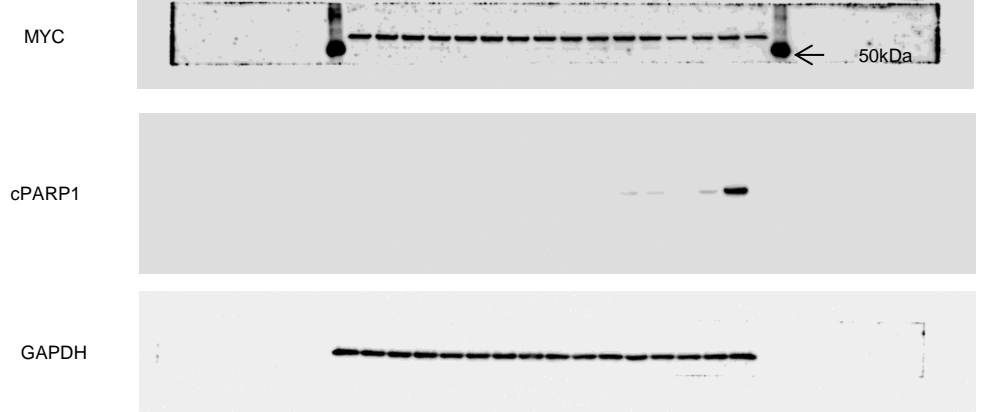

Supplement: Supplementary file 6 — Source Data for Figure 5 [file EMMM-10-e8289-s005.pdf]

Fig 6E

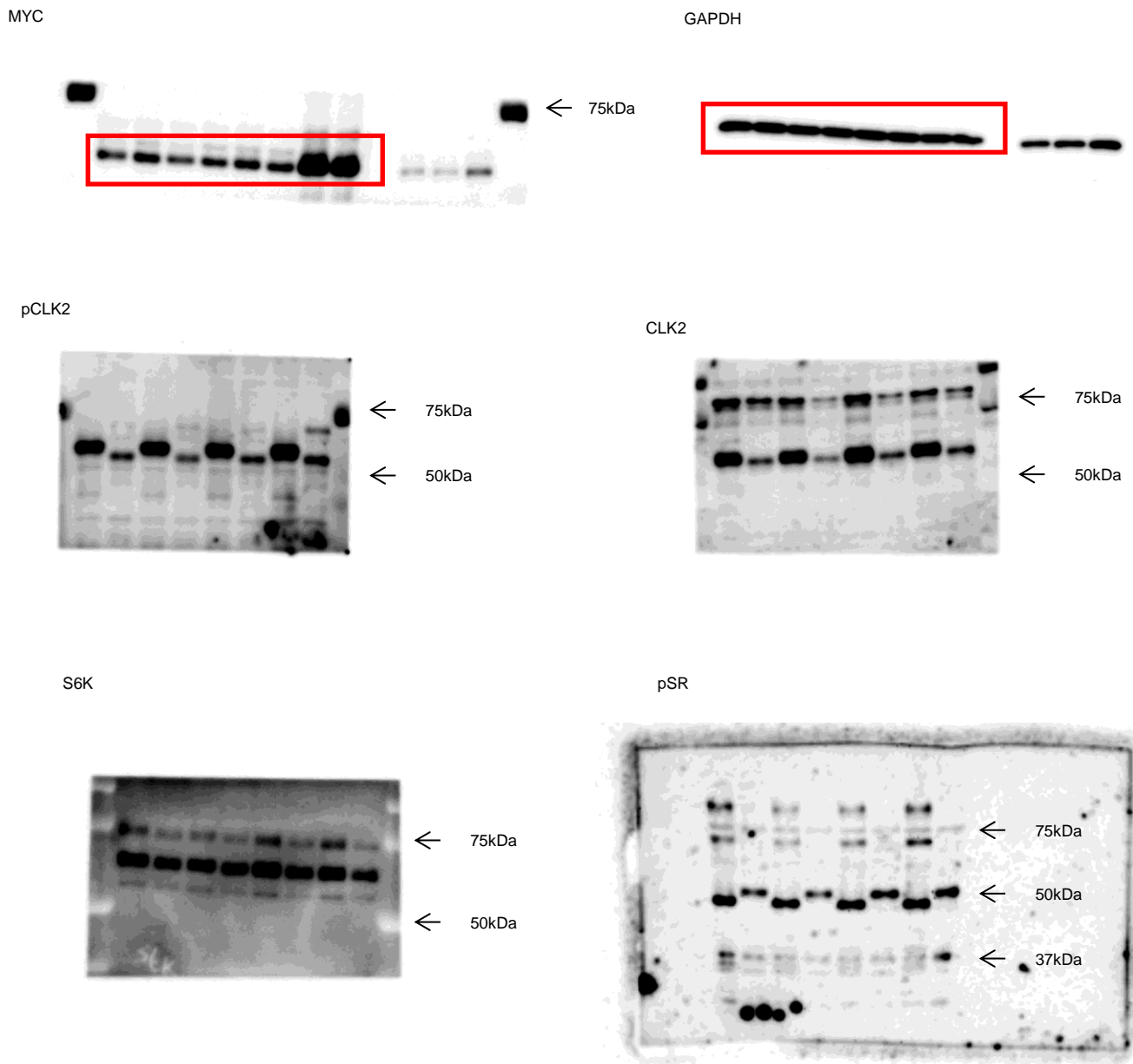

Supplement: Supplementary file 7 — Source Data for Figure 6 [file EMMM-10-e8289-s006.pdf]
